# Changes in the Serum Protein Fractions in Goats after Treatment of Natural Gastrointestinal Parasite Infections

**DOI:** 10.1155/2021/9946519

**Published:** 2021-11-30

**Authors:** Frederika Chovanová, Csilla Tóthová, Róbert Klein, Oskar Nagy

**Affiliations:** Clinic of Ruminants, University of Veterinary Medicine and Pharmacy in Košice, Komenského 73, 041 81, Košice, Slovakia

## Abstract

Gastrointestinal parasitic infections in small ruminants belong to major health problems. The regulation of gastrointestinal infections in goats and the responses developed against them appear to be different from those observed in sheep. In the literature, there is a lack of data on the effect of gastrointestinal parasitic infections on the serum protein profile in goats. Therefore, the aim of the study was to determine the electrophoretic pattern of serum proteins in goats naturally infected with gastrointestinal parasites and to compare the changes in the total serum proteins and serum protein fractions (albumin and *α*_1_-, *α*_2_-, *β*-, and *γ*-globulins) obtained after antihelminthic treatment. Eight adult female goats of the white shorthaired breed from a small dairy goat farm at the age ranging between 3 and 5 years with average body weight 35.4 ± 3.2 kg and body condition score (BCS) from 1.5 to 2.5 were used in the study. The serum proteins in goats were separated into five fractions: albumin, *α*_1_- and *α*_2_-globulins, and *β*- and *γ*-globulins. Significant changes after treatment were found in the relative concentrations of albumin (*P* < 0.01) and *α*_2_- (*P* < 0.05), *β*- (*P* < 0.001), and *γ*-globulins (*P* < 0.01), as well as albumin/globulin ratio (*P* < 0.01). The mean concentration of total serum proteins was, after the antiparasitic treatment, significantly higher. Among the globulin fractions, the *γ*-globulin fraction contributed most significantly to these changes (*P* < 0.001). The results presented in the study suggest a significant effect of antiparasitic treatment in goats on the synthesis of blood serum proteins and on the changes of the proportion of serum protein fractions.

## 1. Introduction

Gastrointestinal nematode infections in small ruminants belong to major health problems in the developing world and are most common in tropical and subtropical regions, but globally, they continue to be a major constraint also for other areas, not only for poor developing countries [1, 2]. The mild climate in countries of Central Europe allows animals to spend most of the year on pasture, where they usually are in contact with infective stages of parasites, which increase the risk of infection [3, 4]. Many literature sources point to the frequent occurrence and high prevalence of gastrointestinal nematodes in small ruminants in many countries and analyse risk factors for infection [5–11]. The gastrointestinal parasitic infections not only affect the health but also indirectly cause huge economic losses in livestock farming due to loss of overall production, reduced animal performance and weight gain, retarded growth, cost of treatment, and mortality [12–14]. Abomasal and intestinal nematode infestation in goats is one of the most impotant diseases worldwide, particularly in those kept outdoors for all or part of the time. Clinical signs may vary from weight loss and reduced milk yield to marked disease with severe watery diarrhoea leading to dehydratation, hypoalbuminemia, and rapid mortality. Infection with intestinal nematodes produces villous atrophy and crypt hyperplasia. The resultant rapid cellular turnover of immature epithelial cells permits loss of fluid and plasma proteins into the intestinal lumen, causing a protein-losing enteropathy [15]. It was described previously that the infections with gastrointestinal parasites in sheep are associated with changes in serum proteins, especially with the decrease of total serum protein and albumin concentrations, and alterations were found also in the serum protein fractions [16, 17]. Data on the effect of gastrointestinal parasitic infections on the serum protein profile in goats are lacking. The worm infections may cause severe damage to host tissue due to direct invasion and gastrointestinal disorders also in goats, but the physiological, biochemical, and immunological reactions to these infections may differ between sheep and goats. Up to now, most data on the evaluation of biochemical changes in the gastrointestinal parasitic infections were recorded mostly in sheep. Therefore, more studies on caprine species are needed to describe the impact of gastrointestinal helminthosis on biochemical parameters and responses of the organism associated with changes in the serum protein profile [18–20]. Protein electrophoresis is the most reliable laboratory method to accurately determine abnormalities in the distribution of serum protein fractions and quantify several serum protein fractions [21]. Even in cases with unchanged total serum protein concentrations, this laboratory technique may be useful to detect possible abnormalities in the serum protein pattern [22]. Therefore, the aim of the present study was to determine the electrophoretic pattern of serum proteins in goats naturally infected with gastrointestinal parasites and to assess the changes in the concentrations of serum protein fractions obtained after anthelmintic treatment.

## 2. Materials and Methods

### 2.1. Ethical Approval

Handling with the animals, sample collection, and preparation were conducted in accordance with the ethical standards and guidelines approved by the Committee of the University of Veterinary Medicine and Pharmacy in Košice on protection of animals used for scientific purposes and complied with the institutional requirements of the Code of Ethics for Scientists (Directive 74/2019/UVLF).

### 2.2. Animals and Sample Collection

Eight adult female goats from a small dairy goat farm including 24 animals in the flock (15 adults and 9 kids) were used in this study. The evaluated animals were of the white shorthaired breed at the age ranging from 3 to 5 years. They were in poor nutritional status, and some of them presented diarrhea. In the faeces of some goats, the presence of released tapeworm proglottids was detected. The average body weight of goats was 31.4 ± 3.2 kg, and body condition score (BCS) ranged from 1.5 to 2.5. Scoring was performed using a BSC ranging from 1 to 5 with 0.5 increments [23]. The animals have been grazed during the day since spring, and during the night, the goats were housed in a barn, where they had free access to meadow hay and water and also received concentrated feed mixture. The fecal and blood samples were obtained during the main pasturing season. Goats on this farm have not been treated with anthelmintics since the beginning of the grazing season.

Fecal samples were collected directly from the rectum of the evaluated goats using plastic gloves into plastic fecal containers. The samples were labelled and then transferred to the the laboratory of the Clinic of Ruminants of the University of Veterinary Medicine and Pharmacy in Košice for the examination of parasitic fecal egg counts. Examination of the samples was carried out within a day. Blood samples for protein analyses were taken twice, the first sampling before treatment of the animals and the second time 3 weeks after treatment to assess the effect of the treatment on changes in the evaluated parameters. The existing helminthosis was treated by oral administration of one dose of albendazole at the dose of 5 mg per kg body weight (Albendavet 1,9%, Slo-Werft). Blood samples were collected by direct puncture of *V. jugularis* into serum gel blood collection tubes with clotting activator, but without anticoagulants (Meus, Piove di Sacco, Italy). After collection, blood samples were left at room temperature to coagulate and then centrifuged at 3000 g for 30 minutes. The separated serum was stored at −20°C until the analyses.

### 2.3. Laboratory Analyses

The standard direct flotation technique described by Taylor et al. [24] was used for the evaluation of eggs of nematodes, cestodes, and coccidian oocysts. The morphological examination of parasitic eggs was performed with light microscopy to identify the eggs of parasites presents. According to standard parasitological criteria described by Sancho [25], a mixed infection with gastrointestinal nematodes and tapeworms was detected. The following types of eggs were identified: *Strongylida*, *Strongyloides*, *Trichuris*, and *Moniesia* spp.

The serum was used for the determination of the concentrations of total serum proteins and protein fractions. Total serum proteins were estimated by the biuret method using commercially available diagnostic kits (Randox, Crumlin, United Kingdom) on an automated chemistry analyzer Alizé (Lisabio, Poully en Auxois, France). Agarose gel electrophoresis was performed with commercial diagnostic kits Hydragel 7 Proteine (Sebia Corporate, Lisses, Evry Cedex, France) using an electrophoresis system Hydrasys (Sebia Corporate, Lisses, Evry Cedex, France) according to the procedure described by the manufacturer.

After electrophoresis, the stained gels were scanned using the densitometry optical scanning system Epson Perfection V700 (Epson America Inc., USA) and evaluated according to the principles of light transmission through the stained gel and convertion into an optical density curve. The computer software program Phoresis 5.50 (Sebia Corporate, France) was used to visualize the bands as peaks (electrophoretogram). The protein fractions were identified by visual inspection according to the midpoints between the peaks on the electrophoretogram. In the evaluated goats, the following protein fractions were identified on the electrophoretogram: albumin, *α*_1_- and *α*_2_-globulins, *β*-globulins, and *γ*-globulins. According to the obtained optical density, the area under each peak was evaluated and the relative concentrations (%) of individual zones were calculated as percents of the total serum proteins. Consequently, the absolute concentrations (g/l) of each band were derived from percents and quantified from the total serum protein concentrations. The ratios of albumin to globulins (A/G) were also calculated.

### 2.4. Statistical Analyses

For each of the evaluated variable the means and standard deviations were calculated using the statistical software program GraphPad Prism V5.02 (GraphPad Software Inc., California, USA). The distribution of data was evaluated by the Kolmogorov–Smirnov test for normality. Not all the evaluated parameters showed normal distribution. The differences between the sample collections (before and after treatment) were tested using a paired t-test for normally distributed data and the Wilcoxon test in case of nonnormally distributed data. Differences were considered significant at *P* < 0.05 level of probability.

## 3. Results

The serum proteins in goats were separated into five fractions: albumin, *α*_1_- and *α*_2_-globulins, *β*-globulins, and *γ*-globulins. As presented in Table 1, the analysis of the relative concentrations of albumin showed a significant decrease of values after the antiparasitic treatment (*P* < 0.01). While the mean values of *α*_1_-globulins were not significantly different before and after treatment of animals, significant alterations between the sample collections were found in the *α*_2_-globulins, showing a significant decrease of the relative values after treatment (*P* < 0.05). An opposite trend was observed in *β*- and *γ*-globulins, with a significant increase of mean concentrations in both globulins after the antihelminthic treatment (*P* < 0.001 and *P* < 0.01, respectively). The A/G ratios decreased and were significantly lower after the treatment (*P* < 0.01).

As presented in Table 2, the mean concentration of total serum proteins after the antiparasitic treatment increased significantly by more than 10 g/l when compared to the mean value before treatment (*P* < 0.001). The analysis of the absolute concentrations of protein fractions showed, with the exception of albumin, significant changes in the values after deworming of the animals. The absolute concentrations of albumin after treatment were nonsignificant though were slightly higher than before the treatment. In the concentrations of *α*_1_- and *α*_2_-globulins, a significant increase of values was found after the antihelminthic treatment (*P* < 0.001 and *P* < 0.05, respectively). Similarly, the absolute concentrations of *β*-globulins increased significantly after treatment (*P* < 0.001). The most marked increase in the absolute concentrations among the globulin fractions was observed in the concentrations of *γ*-globulins. Their values after treatment increased by more than 7 g/l compared to pretreatment concentrations (*P* < 0.001).

**Figure 1:**
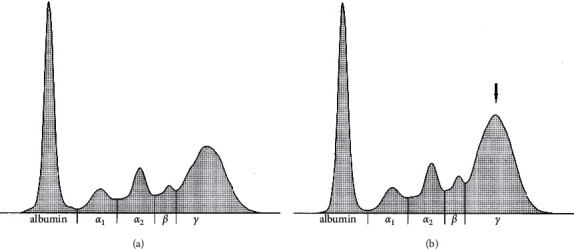
The representative electrophoretograms before (a) and after (b) antiparasitic treatment of goat showing the serum protein fractions of albumin and *α*_1_-, *α*_2_-, *β*-, and *γ*-globulins (*X*-axis- protein fractions).

Changes in the relative proportions of individual serum protein fractions before and after treatment are presented on representative goat electrophoretograms (Figure 1) and indicate a dominant difference, especially visible in the *γ*-globulin fraction (arrow in Figure 1(b)).

## 4. Discussion

Small ruminants, especially grazing animals, are infected usually with more than one species of gastrointestinal parasites worldwide [4]. Not only gastrointestinal helminths but also *Eimeria* spp. are routinely identified in fecal samples and have an important role in mixed gastrointestinal infections [26]. The regulation of gastrointestinal infections in goats and the immune responses developed against them appear to be different from those observed in sheep, but only few studies investigated these reactions in goats and the most of them were conducted in animals infected with *Haemonchus contortus* [18, 27]. Gastrointestinal parasites induce not only morphological changes in the gut lumen, tissue injury, and reduction in the feed digestibility but also a series of hematological and biochemical alterations [28–33]. The damage of gastrointestinal mucosa is accompanied with the leakage of proteins to the gastric lumen resulting in protein-losing enteropathy and decreased concentrations of blood serum proteins [34]. Fernandez et al. [16] observed in goats infected with gastrointestinal parasites, including *Trichostrongylus* spp., *Oesophagostomum* spp., and *Eimeria* spp., decrease in the concentrations of total serum proteins and albumin. A significant decrease of total serum proteins in goats with gastrointestinal parasitic infections was recorded also by Moudgil et al. [35]. This finding was attributed to protein-losing gastroenteropathy and malabsorption of proteins from damaged intestinal mucosa. Intense hypoproteinemia and hypoalbuminemia were obtained also in goats naturally infected with *Haemonchus contortus* [29, 36] or goats with severe parasitic gastroenteritis [32]. An increase of total serum protein concentrations in goats with endoparasites was observed by Hassan et al. [37] on days 14 and 28 after treatment compared to the pretreatment values. Similarly, Alam et al. [38] recorded in goats with clinical fascioliasis the increase of total serum proteins on day 30 of treatment. In our study, the lower concentrations of total serum proteins obtained in goats before antiparasitic treatment were accompanied by the increase of values after treatment, which suggest the improvement of appetite, increased feed intake, and reduction of intestinal nutrient losses. However, without in-depth analysis of individual serum protein fractions, it is not possible to determine which protein fraction contributes to changes in total protein concentrations. As is presented in our study, the recorded increase of serum protein concentrations after antihelmintic treatment may be associated with the increase of *β*- and, predominantly, *γ*-globulins as part of the humoral immune response to overcome the infection. Heavy infections can cause severe illness and may lead also to the loss of albumin from the blood vessels due to its selective loss and increased fractional catabolic rate [24]. Another cause of hypoalbuminemia may be its impaired synthesis by the liver [39]. Albumin belongs to the group of negative acute-phase proteins because of its decreased concentrations in response to injury and inflammation [40]. Thus, the lower concentrations of albumin in the infected animals may be a result of acute-phase response as well due to gastrointestinal parasitic infection. Lower concentrations of albumin were also reported by Bandhaiya et al. [41] in goats with high worm burden, and the egg count in the infected animals highly correlated with the concentration of albumin. Similarly, reduced albumin concentrations were found by Qamar and Maqbool [42] in goats with haemonchosis. Albumin values in the animals treated with various types of antihelmintics were markedly higher on day 28 after treatment. Toma et al. [43] recorded in calves infected with gastrointestinal parasites an increase of albumin concentrations on days 90 and 120 after treatment using 3.5% ivermectin with fluazurol, neocidol, and thiazoline. The concentrations of albumin after the treatment with 3.16% ivermectin and cypermetrine decreased on day 90 after treatment and stayed stable till day 120 after treatment. Although the relative proportion of albumin values in our study decreased after treatment (caused by the marked increase of *γ*-globulin fraction), the absolute values showed no significant slight increase of mean value after treatment. In the concentrations of *α*_1_-globulins, a significant increase of absolute values was observed in goats after the antihelmintic treatment compared to values recorded before treatment. The *α*_1_-globulin fraction comprises many important acute-phase proteins, including alpha_1_-antitrypsin, *α*_1_-acid glycoprotein, serum amyloid A [22]. Thus, the higher concentrations of *α*_1_-globulins in the infected goats may be related to the increased production of some acute-phase proteins due to tissue injury and inflammation caused by the parasites. Higher *α*_1_-globulin concentrations were observed in sheep with naturally acquired gastrointestinal nematode infections [17]. In goats, *Haemonchus contortus* infection was found to cause markedly increased *α*-globulin values [36]. However, the effects of a mixed gastrointestinal parasitic infection in goats on the *α*-globulin fractions, as well as the alterations after treatment, are not completely understood. The increase of *α*_1_-globulin concentrations observed in our study in goats after treatment may suggest the continuation of the acute-phase response or insufficient treatment response in some animals. It was described in horses that antihelmintic treatment may result in a localized inflammatory response characterized also by increased concentrations of some acute-phase proteins [44]. An increase of values after treatment was observed in our study also in the *α*_2_-globulins. This fraction contains some other acute-phase proteins, including haptoglobin, ceruloplasmin, *α*_2_-microglobulin, *α*_2_-macroglobulin, and *α*_2_-lipoprotein [21]. Increased concentrations of haptoglobin and ceruloplasmin were observed by Ulutaş et al. [45] in goats with mixed gastrointestinal infections of nematodes and liver trematodes (*Trichuris* spp., *Trichostrongylidae* spp., and *Fasciola* spp.), but further studies of the clinical importance of the increases of this fraction in relation to the evaluation of treatment efficacy of antihelmintic therapy in goats should be made. Similar to *α*-globulins, the *β*-globulin fraction showed an increase of relative and absolute mean values of *n* goats after the antihelmintic treatment. Some important proteins migrate into this fraction such as complement, C-reactive protein (CRP), and transferrin, as well as ferritin [22]. The activation of the complement pathway is one of the first nonspecific innate defense responses to the infections with parasitic helminths, resulting in increased synthesis of chemotactic peptides C3a and C5a, which mobilize eosinophils to the site of infection [46, 47]. The increase of the *β*-globulin fraction after antiparasitic treatment may reflect the increase of some components of the complement system, suggesting the continuation of inflammatory responses after therapy. Nearly all helminths invade the tissues of the gastrointestinal tract and consequently may cause the initiation of the acute-phase response with the increase of acute-phase protein concentrations. Although CRP in small ruminants is not the diagnostically important, acute-phase protein with only a minor increase during the inflammatory responses, its marked increase was observed in some disease conditions. A significant increase of C-reactive protein values was found by El-Deeb et al. [48] in goats with contagious caprine pleuropneumonia. The alterations of CRP concentrations in goats infected with gastrointestinal nematodes are not yet studied. Another possible cause of hyper-*β*-globulinaemia in the infected goats is the increased production of some immunoglobulins, especially IgA and IgM, directed against the invading parasites, as these immunoglobulins may migrate into the *β*-zone [49]. Therefore, further analyses are needed to establish which proteins are responsible for the elevation of *β*-globulins in goats after antihelmintic treatment.

Not only cell-mediated immunity but also humoral immune responses are initiated against nematode infections in the infected animals [50]. The elevated synthesis of *γ*-globulins is the main component of humoral immune responses to overcome the infection and is the cause of higher *γ*-globulin values in goats in our study. An increase in the concentrations of *γ*-globulins was observed also in sheep and lambs with nematode infections, which was associated with the manifestation of humoral immune response against the parasitic infection [51, 52]. Similarly, higher concentrations of *γ*-globulins were reported by Alam et al. [29] and Diogenes et al. [36] in goats infected with *Haemonchus contortus* and by Jesse et al. [32] in goats with mixed gastrointestinal nematode infections. The results presented in our study showed an increase of *γ*-globulin concentrations after treatment, which was probably associated with late immune response against the parasitic infection. Higher immune response after antihelmintic treatment using ivermectin has been reported previously and was related to massive release of antigens due to synchronous death of parasites, while the immune changes seem to depend on the type of parasite and host species [53, 54]. Similarly, it was stated in horses that the antihelmintic treatment using fenbendazole and moxidectin may upregulate the immune response [55]. Albumin/globulin ratio was also affected in the evaluated animals. After antihelmintic treatment, we recorded a decrease of A/G ratio, and this was associated with more marked increased globulin fractions and decreased albumin fraction after treatment.

## 5. Conclusions

In conclusion, up to now, only few data on the evaluation of biochemical changes in gastrointestinal parasitic infections have been presented in goats. The results recorded in the study suggest a significant effect of gastrointestinal parasitosis and antiparasitic treatment in goats on the synthesis of blood serum proteins and the proportion of serum protein fractions. They were associated with a marked decrease of albumin and the increase, especially, of the *γ*-globulin fraction. In the absolute concentrations of protein fractions, an increase was observed in the values of *α*-, *β*-, and *γ*-globulins. However, further studies using larger animal groups and more frequent sample collections in a longer period after the antiparasitic treatment would be helpful to yield reliable results.

## Figures and Tables

**Table 1:** Differences in the relative concentrations of serum protein fractions (%) and albumin/globulin ratio (A/G) in goats before (BT) and after (AT) antiparasitic treatment (mean ± SD).

Variables	Time of analysis		*P* value
	BT	AT	
Albumin	41.6 ± 6.01	36.9 ± 6.57	<0.01
*α*_1_-globulins	7.9 ± 0.88	7.7 ± 1.12	n.s.
*α*_2_-globulins	13.1 ± 1.06	12.5 ± 1.20	<0.05
*β*-globulins	5.0 ± 0.69	5.6 ± 0.78	<0.001
*γ*-globulins	32.5 ± 6.53	37.2 ± 7.09	<0.01
A/G	0.7 ± 0.19	0.6 ± 0.19	<0.01

*P* value- significance of the differences in means; n.s.- not significant.

**Table 2:** Differences in the concentrations of total serum proteins (TPs, g/l) and absolute values of protein fractions (g/l) in goats before (BT) and after (AT) antiparasitic treatment (mean ± SD).

Variables	Time of analysis		*P* value
	BT	AT	
TP	74.0 ± 5.56	85.0 ± 5.47	<0.001
Albumin	30.6 ± 3.87	31.1 ± 4.09	n.s.
*α*_1_-globulins	5.8 ± 0.73	6.5 ± 0.82	<0.001
*α*_2_-globulins	9.7 ± 0.50	10.7 ± 0.90	<0.05
*β*-globulins	3.7 ± 0.64	4.8 ± 0.80	<0.001
*γ*-globulins	24.2 ± 6.14	31.9 ± 7.61	<0.001

*P* value- significance of the differences in means; n.s.- not significant.

## Data Availability

The datasets generated and/or analysed during the current study are available from the corresponding author on reasonable request.
